# Gender shapes the formation of review paper collaborations in microbiology

**DOI:** 10.1098/rspb.2023.0965

**Published:** 2023-07-12

**Authors:** Rachel M. Wheatley, Lois Ogunlana

**Affiliations:** Department of Biology, University of Oxford, Oxford, UK

**Keywords:** gender, scientific publishing, bibliometric analysis, reviews, collaboration

## Abstract

Women are underrepresented in senior academic positions within microbiology globally. Studies show that gender bias affects the progression of women in academia, but there is evidence that improving conscious awareness of bias can improve equity in this regard. Here we analyse the publication data associated with review articles within the microbiology field to investigate the statistical associations with author gender. We analyse the data from review articles published between 2010 and 2022 in three leading microbiology review journals: *Nature Reviews Microbiology*, *Trends in Microbiology* and *Annual Review of Microbiology*. We find a significant association between the gender of the lead author and the gender of co-authors in multi-author publications. Review articles with men lead authors have a significantly reduced proportion of women co-authors compared to reviews with women lead authors. Given the existing differences in the proportions of men and women in lead author positions, this association may have important consequences for the relative visibility of women in microbiology, along with negative impacts on scientific output relating to reduced collaboration diversity.

## 1. Introduction

Senior microbiology research positions within academia often show a significant underrepresentation of women. The percentage of doctorates awarded to women in the life sciences is over 50%, but the number of women in postdoctoral and tenure-track positions is less than 40% and 30%, respectively [1,2]. Only 18% of full professors in biology-related fields are women [3]. Numerous studies have shown how gender bias exists in professional evaluations [4,5], promotions [6], grant proposal success [7,8], salaries [9,10] and the acknowledgement of contributions to work [11]. Improving conscious awareness of where these inequalities exist is a first step towards improving equity in this regard [12]. The quantitative analysis of data associated with academic publications (i.e. bibliometric analyses) is one way in which this can be achieved.

Bibliometric analyses often focus on primary research. These types of analysis have revealed differences in manuscript submission outcomes [2], citation metrics [13,14] and the volume of self-citations [15] associated with author gender. Such analyses typically neglect review articles, yet, review articles can be considered a metric of who is an expert in the field. Authoring review articles can increase the profile, visibility and citations of a researcher.

Although single-author reviews are not uncommon, reviews in microbiology typically have multiple authors. To this end, review articles provide the opportunity to work collaboratively on an intellectual project, and the possibility to transcend some of the logistical barriers imposed on laboratory-based research. Studies of gender or diversity within groups working on collaborative tasks has shown that mixed-gender or otherwise diverse teams can produce better outputs [16–18] and higher-quality science [19]. Multi-author reviews can also provide senior researchers with the opportunity to contribute to the career development of their juniors. For example, sharing expertise and forming collaborations with other scientists during the review writing process may expand networks and increase the profiles of researchers in their field. This outlines the importance of review publications in academic communities, and the significance of multi-author collaborations. But what decides how collaborations form? Researchers choose collaborators based on expertise, but also social factors such as existing working structures or personal relationships play a role [20,21]. Homophily is the principle that similarity breeds connection between individuals [22], and gender homophily is the principle that individuals assort non-randomly with respect to gender. Due to this social structuring, it is possible that gender plays a role in the assembly of review collaborations [22].

In this study we carry out a bibliometric analysis of microbiology review articles published between 2010 and 2022 in three leading microbiology review journals (*Nature Reviews Microbiology*, *Trends in Microbiology* and *Annual Review of Microbiology*) to investigate the statistical associations with authorship gender. We investigate whether gender may play a role in the formation of microbiology collaborations by investigating whether there is an association between the gender of the lead author and the gender of co-authors in multi-author reviews. Given the existing differences in the proportions of men and women in lead author positions, an association may have important consequences for the relative visibility and career progression of women in microbiology, along with potential impacts on scientific output relating to reduced collaboration diversity [16–19].

## 2. Results and discussion

Publication data was downloaded from Web of Science for review articles published between January 2010 and June 2022 in *Nature Microbiology Reviews*, *Trends in Microbiology* and *Annual Review of Microbiology*. The gender of authors was inferred from first names using Gender API, a social media-informed classification algorithm. This approach was used to infer presenting gender based on first names and has been used extensively in work examining gender in authorship of academic articles (e.g. [2,13,23,24]). This produced a dataset of 1857 review papers with inferences on author gender (electronic supplementary material, table S1). This dataset had a total of 5680 authors and a median of three authors per paper. All references to author gender (i.e. woman/man) in this study use inferred gender. We recognize the limitations of this approach. Inferred gender is distinct from the gender(s) that an individual may identify as, is restricted to a binary mode of inference (i.e. is not inclusive of non-binary individuals), and left a category of unknown inferences that were excluded from the downstream analysis.

**Figure 1.**
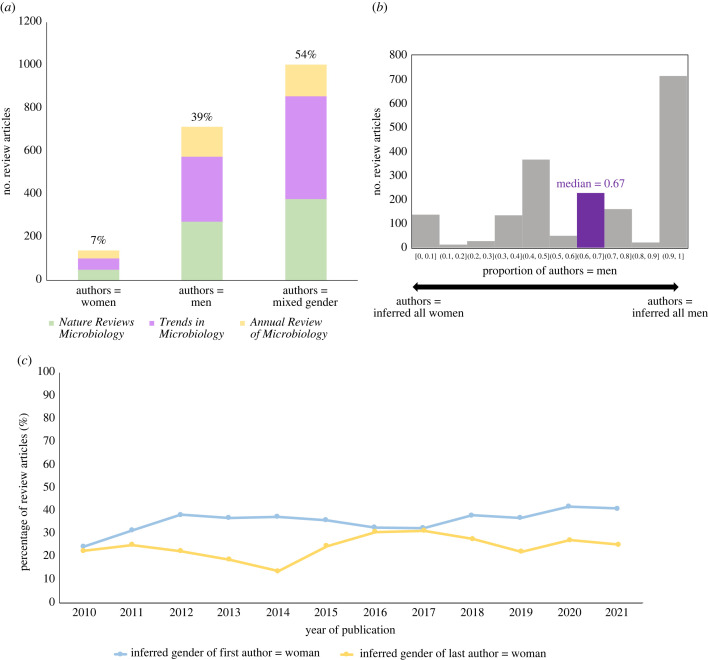
Overview of dataset. (*a*) Number of publications split by inferred gender of author list (authors = women, authors = men, authors = mixed gender) and journal (*Nature Reviews Microbiology*, *Trends in Microbiology*, *Annual Review of Microbiology*). Percentage rounded to 0 d.p. This number includes single author reviews, which account for 199/1857 publications. (*b*) Histogram showing the inferred gender of publication author lists, as 0 = all authors inferred women and 1 = all authors inferred men. The median for this dataset is 0.67 (highlighted in purple). (*c*) Change over time in the proportion of women in first author (blue line) and last author (yellow line) positions.

We find there are over five times as many review articles published with author lists that were inferred to consist of exclusively men compared to exclusively women (figure 1*a,b*). Review articles with author lists that consist of exclusively men accounted for 39% of papers in the total dataset, compared to only 7% for exclusively women, and 54% for mixed gender (figure 1*a*). These numbers include single author reviews, which account for 199/1857 publications.

Author positioning practices can be used to infer leadership roles within publications [25]. In microbiology, as in other fields of biology, it is traditionally assumed that the first author has made the most contributions and the last author is the most senior scientist or principal investigator [26]. Although there are some regional variations in the norms of authorship designation [27], by positioning conventions, the first or last named author should generally capture the majority of authors who have led or initiated a review article.

A total of 36% of reviews had women first authors. The proportion of publications with women first authors has increased over the last 10 years. Rising from 25% of reviews in 2010 to 41% of reviews in 2021 (figure 1*c*), with the sharpest rise occurring between 2010 and 2012. The proportion of publications with women last authors has fluctuated, but from end points remained largely unchanged (figure 1*c*). Women were last authors on 23% of reviews published in 2010 and 25% of reviews published in 2021 (figure 1*c*). Overall a total of 24% of reviews had women last authors.

### (a) Gender influences microbiology review collaborations

We wanted to investigate the relationship between the genders of the lead- and co-authors in multi-author reviews (≥ three authors). Multi-author reviews (≥ three authors) accounted for 1066 publications in our dataset. Due to authorship positioning practices, the first or last named author should generally capture the majority of ‘lead authors’ (i.e. individuals most likely to have assembled publication contributions) for a review collaboration. To this end, we assessed the relationship between the inferred gender of first or last author with the inferred gender of co-authors on a publication.

**Figure 2.**
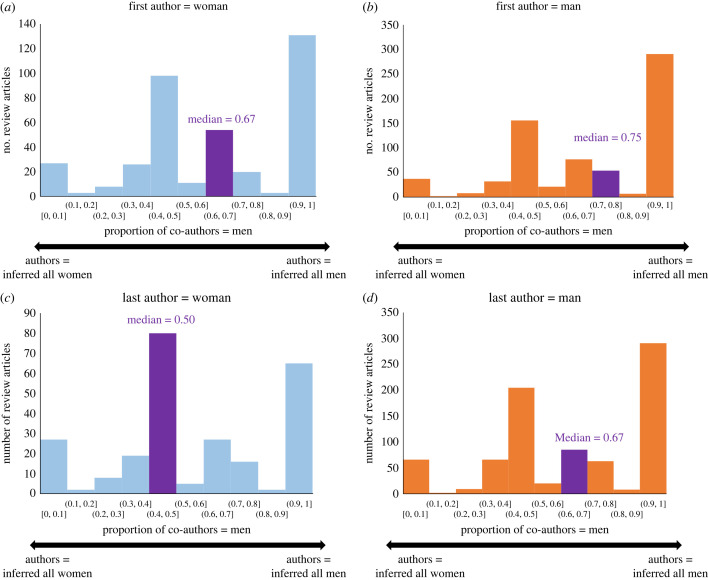
Gender of co-authors by inferred gender of first or last author, shown for (*a*) publications with women first authors, (*b*) publications with men first authors, (*c*) publications with women last authors and (*d*) publications with men last authors. The bar containing the median is highlighted in purple and median values are annotated on plots. Proportion of co-authors is shown on a scale where 1 = all authors inferred men and 0 = all authors inferred women.

We find a significant association between the inferred gender of the lead author and the inferred gender of co-authors in multi-author reviews (figure 2, table 1). On average 75% of co-authors were men when the first author was a man, compared to 67% of co-authors when the first author was a woman (Mann–Whitney, *p* < 0.01 two-tailed) (figure 2*a,b*, table 1). On average 67% of co-authors were men when the last author was a man, compared to 50% of co-authors when the last author was a woman (Mann–Whitney, *p* < 0.01 two-tailed) (figure 2*c,d*, table 1). This trend was observed across all three journals (table 1). We considered that as the number of authors on a publication increases, the probability of having mixed co-authorship should also increase. We confirmed that the size of the author list was not significantly different between publications with men or women lead authors (Mann–Whitney, *p* < 0.05 two-tailed). While due to author positioning conventions it is typically assumed that both the first or last named author may play an important role in leading the review, it is also typically assumed that the last author may be the most senior scientist or principal investigator [26], and as such, could be playing a more defining role over the teams composition. In line with this, it is interesting to note these differences in co-author compositions between first and last author gender (figure 2, table 1), along with the fluctuations in women in first or last author positions over time (figure 1).

**Table 1.** Inferred gender of first and last author and inferred gender of co-authors. On average 75% of co-authors were men when the first author was a man, compared to 67% of co-authors when the first author was a woman (Mann–Whitney, *p* < 0.01 two-tailed). On average 67% of co-authors were men when the last author was a man, compared to 50% of co-authors when the last author was a woman (Mann–Whitney, *p* < 0.01 two-tailed). For each journal and the total, the author row containing the highest proportion is highlighted in blue.

journal	inferred gender of first author	median proportion of co-authors = men	total papers	inferred gender of last author	median proportion of co-authors = men	total papers
*Annual Review of Microbiology*	woman	0.50	52	woman	0.50	38
	man	0.75	83	man	0.67	97
*Nature Reviews Microbiology*	woman	0.67	140	woman	0.60	84
	man	0.75	271	man	0.67	327
*Trends in Microbiology*	woman	0.67	189	woman	0.50	129
	man	0.67	331	man	0.67	391
**Total**	**woman**	0.67	381	**woman**	0.50	251
	**man**	0.75	685	**man**	0.67	815

**Figure 3.**
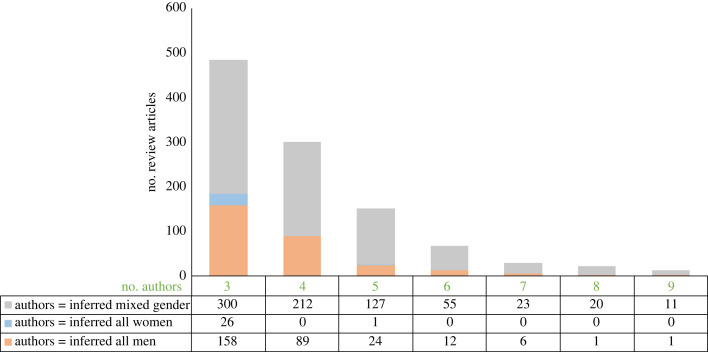
Prevalence of single gender teams in multi-author reviews (≥ three authors). Number of review articles shown by the number of authors and inferred gender of teams (all men (orange), all women (blue) or mixed gender (grey)). The table below indicates the numbers of review articles in each category.

We finally wanted to investigate the prevalence of single gender teams (i.e. when author lists are inferred as all men or all women) specifically in this subset of multi-author reviews (≥ three authors) (figure 3). As expected, we see that as the size of the author list increases, the proportion of mixed gender teams increases (figure 3). All women teams accounted for a total of 27 review papers, of which 26 were three author papers and 1 was a five author paper. All men teams accounted for a total of 291 review papers, of which 158 were three author papers, 89 were four author papers, 24 were five author papers, 12 were six author papers, 6 were seven author papers, 1 was an eight author paper and 1 was a nine author paper.

The prevalence of single-gender teams could be expected to vary, if based on random assortment with respect to gender, based on the proportion of researchers of a single gender. In the simplest scenario—if the proportion of men and women within a field is 50 : 50 and teams of two form, you could expect 25% of teams to be two women, 25% of teams to be two men, and 50% of teams to be mixed gender. The proportions of men and women vary in the life sciences, particularly across career stage [1,2]. The proportion of women in postdoctoral positions has been estimated at approximately 40% [1,2], and we take this as an average career stage contributing to multi-author review articles. We use these values (40% women versus 60% men) to calculate expected numbers of single-gender teams if based on random assortment with respect to gender. We find that the observed number of all-men teams (figure 3) is significantly higher than that expected across all team sizes (one-tailed paired *t*-test, *p* < 0.001). On the other hand, we find that the observed number of all-women teams (figure 3) does not differ significantly from the expected (one-tailed paired *t*-test).

As an example of this comparison, if we look at reviews with three authors, simple probability tells us that the expected proportions of single gender teams if based on random assortment with respect to gender would be 21.7% (teams of three men; 0.6 × 0.6 × 0.6) and 6.4% (teams of three women; 0.4 × 0.4 × 0.4). Comparatively, 32.6% of our three author review papers were inferred to be written by teams of three men, and 5.4% written by teams of three women (figure 3). This difference can become more pronounced in larger teams. For review articles with seven authors we found that 20.7% of papers (6/29) in our dataset were inferred to be written by teams of seven men (figure 3), compared to the 2.8% that could be expected based on random assortment of authors via author gender proportion at 60%. We note that the observed value could be expected via this simple probability if the gender proportion of men was >80%.

Overall our findings suggest that gender is a factor that influences review collaboration formation in microbiology and that lead authors who are men are more likely to invite men co-authors to collaborate on review publications. This agrees with a number of studies across disciplines that shows collaborators assort non-randomly with respect to gender [27–33], and supports that this gender homophily may be pervasive in microbiology review publications. In terms of impact, review articles can be considered a metric of who is an expert in the field, and authoring review articles​ can increase the profile, visibility and citations of a researcher. As such, given the existing differences in the proportions of men and women in lead author positions, this association may have important consequences for the relative visibility, profile and citations of women in microbiology. Furthermore, studies of gender or diversity within groups working on collaborative tasks show that mixed-gender or otherwise diverse teams can produce better outputs [16–18] and higher-quality science [19]. This suggests that reducing homophily within review collaborations would also be beneficial for scientific output [16–19].

## 3. Conclusion

In this study we carry out a bibliometric analysis of review articles in microbiology published between 2010 and 2022 in three leading microbiology review journals to investigate the statistical associations with author gender. Review articles can be considered a metric of experts in the field and can increase the profile and visibility of a researcher. They also provide the opportunity to work collaboratively on an intellectual project, and the possibility to transcend some of the logistical barriers imposed on laboratory-based research. The key finding of this work was that multi-author reviews with men as lead authors have a significantly reduced proportion of women co-authors compared to reviews with women as lead authors. Furthermore, multi-author reviews published by all men teams are common. Taking postdoctoral researchers as the average career stage contributing to reviews, we show that all men teams are more common than would be expected if teams assembled randomly with respect to gender. Given the existing differences in the proportions of men and women in lead author positions, this may have important consequences for the relative visibility and progression of women in microbiology, and this homophily may have negative impacts on the outputs produced in collaborations [16–19]. Our findings point to an underrepresentation of women authors in microbiology reviews, as has previously been shown to be the case in the publication of primary research [2]. This supports the need for journals, editors, and researchers to consider the processes underlying the invitation or proposal of a review and the assembly of review team collaborations.

## 4. Methods

### (a) Data collection and gender inference

Full article information was downloaded from Web of Science for all review articles published between January 2010 and June 2022 in *Nature Reviews Microbiology, Trends in Microbiology* and *Annual Review of Microbiology*, with between one and ten authors. These three journals were chosen through a combination of impact factor evaluation and consensus-based discussion about the long-term professional impact of publication in these journals. This produced a dataset of 2025 papers. The gender of authors was inferred from first names using Gender API [34], a classification algorithm that uses self-reported gender from social media data. The data returned from Gender API included name, inferred gender (as ‘male’, ‘female’ or ‘unknown’), the number of instances the gender associated with the name was reported within the social media database, and a confidence value of the gender inference (ranging from 0.5 to 1.0) based on this number of instances and the variability within them. Low accuracy assignments (below 0.7), unknowns and instances where author first name was not provided in the author list were removed from the dataset. This produced a dataset of 1857 review papers with inferences on authorship gender (here referred to as woman/man) (electronic supplementary material, table S1). Although this approach to infer gender from first name is used extensively in work examining gender in authorship of academic articles (e.g. [2,13,23,24]), we recognize its limitations. Inferred gender based on first names is distinct from the gender(s) that an individual may self-identify as, is restricted to a binary mode of inference, and leaves a category of unknown gendered individuals who were downstream excluded from analysis. We are aware that names from certain countries (e.g. western European countries) are overrepresented in the Gender API database.

### (b) Data analysis

Author inferred genders were converted into numerical (1 = man, 0 = woman), and this was used to compare gender of co-authors within the author list of a paper. Publications with three or more authors were used to investigate the relationship between the inferred gender of the lead author and the inferred gender of co-authors in multi-author reviews. Data were tested for distribution normality using a Kolmogorov–Smirnov test, and a non-parametric test was used to compare group distributions (Mann–Whitney, two-tailed), following [33]. Statistical analyses were carried out in R [35].

### (c) Supplementary information

Table S1 of the electronic supplementary material presents details of the dataset of 1857 review articles.

## Data Availability

The data are provided in the electronic supplementary material [36] and available online via Web of Science.
